# A Case Report of Recurrent Glomerulonephritis 27 Years After Renal Transplant

**DOI:** 10.7759/cureus.23041

**Published:** 2022-03-10

**Authors:** Ariana R Tagliaferri, Katherine Stephens, Sewar H Abuarqob, Michael Maroules

**Affiliations:** 1 Internal Medicine, St. Joseph's Regional Medical Center, Paterson, USA; 2 Hematology and Oncology, St. Joseph's Regional Medical Center, Paterson, USA

**Keywords:** immunosuppressants, acute transplant rejection, end stage renal disease, renal failure, chronic transplant rejection, membranoproliferative glomerulonephritis

## Abstract

Herein we present the case of a patient who was diagnosed with membranoproliferative glomerulonephritis and underwent renal transplant 27 years prior to presentation with new kidney failure. Although our patient did not undergo renal biopsy, it is our thought that she developed recurrent membranoproliferative disease, as she was well maintained on immunosuppressants and steroids for many years. This case is unique, because she was outside of the typical window for both chronic rejection and recurrent disease. This case also raises awareness of the utility of renal biopsy to differentiate these two conditions, which allows physicians to treat accordingly.

## Introduction

Membranoproliferative glomerulonephritis (MPGN) is a pattern of glomerular injury characterized by endocapillary proliferation, mesangial hypercellularity, and capillary-wall remodeling [1]. It is a combination of a nephrotic and nephritic syndrome, and patients can be asymptomatic or present with a plethora of symptoms including proteinuria, hematuria to rapidly progressive glomerulonephritis [1,2]. The three main types of MPGN are historically defined by the location and appearance of immune deposition visualized on electron microscopy [1,3]. Type I is the most common and consists of subendothelial and mesangial deposits [3]. Type II is characterized by highly osmiophilic dense deposits within the lamina densa of the glomerular basement membrane and is often referred to as dense deposit disease [3]. Finally, type III is characterized by subepithelial and subendothelial deposits [3]. Currently, this classification model has been redefined to describe the mechanism of glomerular injury, including immune-complex-mediated versus complement-mediated glomerulonephritis [1].

Primary glomerulonephritis is one of the leading causes of end-stage renal disease (ESRD) worldwide, with approximately 50% of MPGN progressing to ESRD within 8-10 years after initial presentation [1,4]. Secondary MPGN is caused by systemic diseases, such as hepatitis C, plasma cell dyscrasias, and autoimmune disorders, and typically manifests as type I or III MPGN [1,4]. Patients with ESRD who undergo renal transplantation are at risk for both disease recurrence in the transplanted kidney, and acute or chronic rejection of the transplanted kidney [2,5]. There are new immunosuppressive agents geared toward decreasing acute and chronic rejections, but they have not changed the incidence nor long-term outcomes of either recurrent or de novo glomerulonephritis after renal transplant [5,6]. Clinically, it can be difficult to distinguish between rejection, recurrent glomerulonephritis, or de novo glomerulonephritis, as there are not uniform tools to diagnose or differentiate these [5,6]. For example, tissue is not routinely sent for electron microscopy and immunofluorescence and, additionally, histological changes may be subtle in early stages or recurrent disease [5].

## Case presentation

A 46-year-old Hispanic female with a history of MPGN status post renal transplant 27 years prior to presentation was referred to the emergency department by primary care for an elevated creatinine level of 6.1 mg/dL. The patient had a 30-year history of MPGN due to unknown etiology and developed ESRD two years following initial diagnosis, requiring peritoneal hemodialysis for one year before undergoing renal transplantation. Following transplant, she was maintained on cyclosporine for 10 years, and then transitioned to prednisone and cisplatin; however, exact dosing was unknown. The patient’s creatinine was within normal limits on the aforementioned regimens after transplantation. All medical care were performed in another country and, thus, the rationale for maintenance on this regimen, as well as exact renal function labs are ultimately unknown. The patient was not taking any other medications, including anti-hypertensives. 

**Figure 1:**
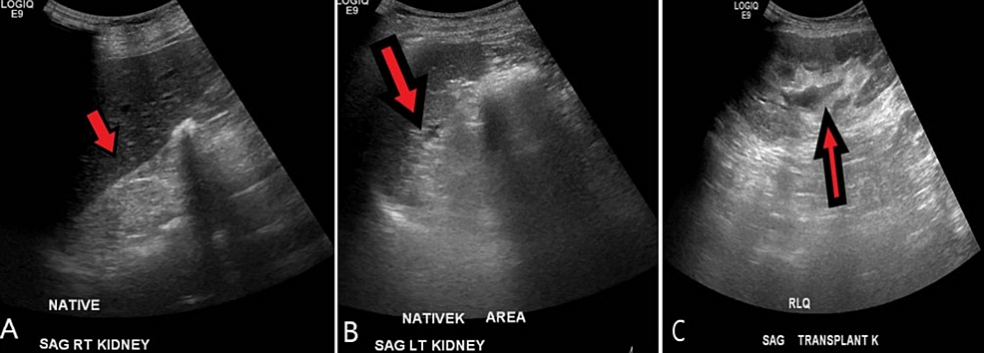
Ultrasound of native and transplanted kidneys with Doppler imaging. A real-time sonography of the kidneys and bladder was performed. The native kidneys are markedly atrophic and difficult to visualize (A: sagittal view of the right native kidney; B: sagittal view of the left native kidney). The transplant kidney measures 12.5 cm in size (C: sagittal view of the transplant kidney in the right lower quadrant).  No hydronephrosis, shadowing stone, or mass lesion is identified. The resistive indices are within normal limits.  Red arrows identify the kidney.

Upon this presentation in the United States, she reported frontal headaches associated with palpitations and exertional dyspnea lasting one week; however, all other review of systems were negative. On arrival, her blood pressure was 190/100 mmHg, for which she was treated with amlodipine 10 mg and labetalol 10 mg without improvement. Her examination was non-contributory. Admission labs were significant for hyperkalemia (5.2 mEq/L), abnormal kidney function (blood urea nitrogen [BUN] 76 mg/dL/creatinine 5.31 mg/dL), and normocytic anemia (hemoglobin 7.4 g/dL, mean corpuscular volume [MCV] 94 fL), and urinalysis was significant for proteinuria (100 mg/dL) with large leukocyte esterase, greater than 100 white blood cells with bacteria present. The reference ranges are as follows: potassium 3.5-5.0 mEq/L; BUN 7.0-23.0 mg/dL; creatinine 0.6-1.3 mg/dL; hemoglobin 12.0-16.0 g/dL; MCV 80.0-100.0 fL. She was admitted to the critical care unit for hypertensive emergency and was treated with a nicardipine drip. Renal ultrasound demonstrated a normal size transplanted kidney without hydronephrosis (Figure 1) and CT of the abdomen without contrast revealed diffuse diverticulosis and constipation without obstruction.

The nephrologist initiated methylprednisolone 1 g and intravenous normal saline, with concerns for transplant rejection versus recurrent MPGN in the transplanted kidney. Urine cultures grew *Escherichia coli*, which was treated with a seven-day course of ceftriaxone. She was transitioned to oral anti-hypertensives (amlodipine 10 mg daily and carvedilol 25 mg twice daily). The patient underwent arteriovenous fistula creation and hemodialysis was initiated via Perm-A-Cath until the fistula matured. Approximately one month later at her inpatient follow-up, her creatinine improved to 3.87 mg/dL. She was scheduled for renal biopsy of the transplanted kidney; however, the patient did not return for further follow-up. 

## Discussion

The true rate of recurrence of MPGN after renal transplant is unknown; however, studies have shown rates between 2.5% and 50% [6]. These discrepancies are due to incomplete biopsy, variable follow-up times and diagnostic criteria, inconsistent reporting practices and thresholds for transplantation [6]. One study showed disease recurrence in approximately 10.3% of patients who underwent kidney transplant for various types of glomerulonephritis, of which 11.8% occurred five years after transplantation, 15.6% after 10 years, and 18.9% after 15 years [6]. All subtypes of glomerulonephritis are at risk of recurring after transplantation; however, those that carry the highest risks are IgA nephropathy, idiopathic membranous glomerulonephritis, focal segmental glomerulosclerosis (FSGS), and MPGN [4]. Interestingly, earlier recurrences of allograft failure occur in patients with subtypes MPGN and FSGS compared to other subtypes, which present most commonly 3-5 years following transplant [4].

Several risk factors have been identified for recurrence such as male sex, younger recipient age, closer HLA matching, low complement levels, presence of monoclonal gammopathy, and living donors who are blood-related to the patient [1,6]. Age was also determined to be an independent factor, where there was a 2% decrease in recurrence for every advancing year of age up until the age of 40 [6].

This makes our case particularly interesting as she presented 27 years after transplantation at the age of 46 years, with adequate renal function during that time course. Additionally, systemic infections, other autoimmune diseases, calcineurin inhibitor toxicities, or acute humoral rejection can lead to the development of transplant glomerulonephritis, or de novo disease; however, these were not present and unlikely to be the cause of her MPGN in the transplanted kidney. 

Chronic organ rejection usually occurs months or years after transplantation and may be referred to as chronic allograft vasculopathy [7]. Several mechanisms play an essential role in the immunopathogenesis of chronic rejection such as chronic inflammation, and humoral and cellular immune reactions [7]. The severity and speed in which the host detects donor tissue as foreign are dependent on the histocompatibility between the donor and recipient [7]. Therefore, HLA-matched grafts survive longer compared to HLA-mismatched grafts [7]. Our patient’s transplantation took place in another country and thus we are not able to determine the type of graft; however, as her tissue was functional for 27 years, it is likely that she may have had a HLA-matched graft. Acute and chronic rejection may be prevented with the combination use of immunosuppressive agents such as T-cell inhibitors (cyclosporine, tacrolimus, sirolimus), antiproliferative drugs (6-mercaptopurine, mycophenolic acid), and anti-inflammatory agents (corticosteroids) [7]. Cisplatin is not commonly used and is also nephrotoxic [7]. Interestingly, this was one of the maintenance medications used in our patient for three years and may have been a contributing factor to her declining renal disease. 

Regardless, patients with declining renal function after transplant must be evaluated for recurrence of MPGN versus chronic kidney rejection [5]. Both conditions can present similarly with hypertensive episodes, proteinuria, hematuria, and increasing BUN/creatinine [5]. Although it is difficult to distinguish between the two diseases pathologically, even for the most experienced pathologists, a renal biopsy is still warranted to differentiate between the two diseases [5]. Therefore, it can only be presumed that the cause of our patient’s declining renal function is due to MPGN, as she did not return for a biopsy.

## Conclusions

Due to inconsistent reporting and lack of confirmative biopsies, the true rate of recurrent MPGN after renal transplantation is unknown. The maximum time frame of recurrence that has been reported is up to 15 years after transplantation, which makes our case unique that our patient’s renal function declined only after 27 years. Additionally, although her hypertension may have increased the risk of chronic rejection, she was also outside of this window and was well maintained on immunosuppressants for many years without changes to her renal function. Thus, it can be questioned if there are other factors related to her declining kidney function such as use of cisplatin, or other risk factors that may have led to her disease state. A renal biopsy would have been confirmative in this circumstance and is suggested for all clinicians to distinguish between the two conditions. 
